# Predicting Microvascular Invasion in Liver Transplant Recipients for Hepatocellular Carcinoma

**DOI:** 10.7759/cureus.75007

**Published:** 2024-12-02

**Authors:** Usman I Aujla, Imran Ali Syed, Kashif Rafi, Ammara Naveed, Ahmad K Malik, Muhammad Yasir Khan, Ihsan Ul Haq, Sohail Rashid, Osama T Butt, Faisal Dar

**Affiliations:** 1 Gastroenterology and Hepatology, Pakistan Kidney and Liver Institute and Research Center, Lahore, PAK; 2 Gastroenterology, Pakistan Kidney and Liver Institute and Research Center, Lahore, PAK; 3 Hepatology, Pakistan Kidney and Liver Institute and Research Center, Lahore, PAK; 4 Adult Gastroenterology, Pakistan Kidney and Liver Institute and Research Center, Lahore, PAK; 5 Hepatopancreatobiliary and Liver Transplant Surgery, Pakistan Kidney and Liver Institute and Research Center, Lahore, PAK

**Keywords:** alpha-fetoprotein, hepatocellular carcinoma (hcc), liver transplant, microvascular invasion, milan criteria

## Abstract

Background: Among primary liver tumors, hepatocellular carcinoma (HCC) is considered the most common hepatic tumor. Liver transplantation is one of the curative treatment options for HCC. However, the risk of HCC recurrence after liver transplantation varies and is influenced by various factors. Microvascular invasion (MVI) is a major factor associated with HCC recurrence after a liver transplant (LT). The study assessed the pre-transplant factors to predict MVI on explant liver specimens.

Methods: The retrospective study included adult LT recipients with HCC on explant specimens to identify pre-transplant predictors of MVI. Univariate analyses, including Mann-Whitney U tests and chi-square tests, were conducted to assess associations between variables and MVI. Logistic regression was employed for multivariate analysis, including variables significant in univariate analysis. Pearson or Spearman correlation coefficients were calculated to examine correlations between continuous variables. Cohen's kappa coefficient was used to measure inter-rater reliability.

Results: Out of 523 LT recipients, 136 (26%) were diagnosed with HCC based on pre-transplant imaging and histopathological analysis of the explanted liver. Descriptive data showed an average age of 54.06 ± 8.16 years (range: 15-70), with a majority being male (76.47%). Hepatitis C (HCV) was the leading etiology (72.8%). Most patients had moderately differentiated grade-II tumors (75.7%) and met the Milan criteria (74.3%). Mean pre-operative alpha-fetoprotein (pre-op AFP) levels were 104.42 ± 308.38 ng/ml. 74.3% were within the Milan criteria. MVI was present in 28.7%. The frequency of MVI among HCCs within vs. outside Milan criteria was not statistically significant (26.73% vs. 34.28% (p = 0.395)). Univariate analysis revealed that pre-op AFP levels (p = 0.001), Child-Turcotte Pugh class (p=0.05), and body mass index (p=0.02) were significantly associated with MVI. Multivariate logistic regression analysis showed that pre-op AFP was the only independent predictor of MVI (OR: 1.006, 95% CI: 1.003-1.008, p < 0.001).

Conclusion: This study not only reinforces the clinical significance of pre-op AFP levels as a simple pre-transplant predictor of MVI in patients with HCC but also advocates for the safety of liver transplantation beyond conventional Milan criteria, promoting extended LT protocols.

## Introduction

The incidence of hepatocellular carcinoma (HCC) is rising across the globe [1]. Among primary liver cancers, HCC is ranked the most common hepatic tumor. Approximately 90% of primary liver tumors are HCCs [2]. HCC is the fourth most common cause of cancer-related mortality across the world [3]. The rising incidence of HCC has huge implications for healthcare infrastructure. Its etiology is multifactorial, but hepatitis B and C viruses are the leading causes of HCC [4]. However, with the rising incidence of steatotic liver disease across the globe, metabolic dysfunction-associated steatotic liver disease is an emerging etiology for HCC [3]. HCC is more common among men with a worldwide male-to-female (M:F) ratio of 2.8:1. Among men, it is the second most common cause of cancer-related mortality [3,5]. The age of onset of HCC varies in different parts of the world. In Europe, North America, and Japan, the age of onset is 60 years, while in Asian and African countries, the age of presentation ranges between 30 years and 60 years [2].

Various histological subtypes of HCC have been described in the literature. These include fibrolamellar, clear cell type, steatohepatitic, scirrhous, macrotrabecular massive, chromophobe, neutrophil-rich, and lymphocyte-rich [6]. It is also classified histologically into well-differentiated, moderately differentiated, poorly differentiated, and undifferentiated subtypes [7]. Among all the tumor markers for HCC, alpha-fetoprotein (AFP) is the best-known tumor marker and has been used universally for decades. Other commercially available tumor markers include des-γ-carboxy prothrombin (DCP) and AFP-L3 [8]. A combination of these biomarkers has been used in various diagnostic and prognostic models like BALAD (bilirubin, albumin, AFP-L3, AFP, and DCP) and GALAD (gender, age, AFP-L3, AFP, and DCP) [9].

HCC can be diagnosed radiologically (contrast-enhanced computed tomography scan, magnetic resonance imaging, and ultrasound) owing to the characteristic radiologic appearance. In rare instances, a biopsy specimen is required to establish the diagnosis. To assess the tumor stage, various staging systems have been devised but the most widely utilized one is the Barcelona Clinic Liver Cancer (BCLC) staging system [10]. This staging system allows careful selection of patients for different treatment options including surgical resection, liver transplant (LT), locoregional therapies (radiofrequency ablation, microwave ablation, percutaneous ethanol injection, trans-arterial chemoembolization (TACE), radio-embolization, etc.) and various systemic therapies. However, with angioinvasion or metastatic disease, the prognosis becomes dismal with limited treatment options [11].

Liver transplantation is considered a curative treatment option for selected groups of patients with HCC. Various selection criteria for liver transplantation are used in clinical practice including Milan, University of California San Francisco (UCSF), Up-to-seven criteria, AFP-French model, and Seoul and Hangzhou criteria but the most widely accepted criterion across the world is the Milan criteria [11]. Following liver transplantation, a detailed histopathological examination of the explant specimen is carried out to determine the tumor type, grade, focality, microvascular invasion (MVI), and size to predict the recurrence risk. Various prognostic models are available to predict the recurrence of HCC post-transplant. The Risk Estimation of Tumor Recurrence After Transplant (RETREAT) score is one such prognostic model to predict the recurrence of HCC [12]. This model incorporates three parameters and assigns points against them. These parameters include AFP levels at the time of LT, a sum of the explants' largest viable tumor diameter number of lesions, and the presence or absence of MVI. A combined score of 5 or more increases the risk of recurrence [13]. Published data suggest that MVI is strongly associated with tumor recurrence and hence impacts survival after liver transplantation [13]. We conducted this study to determine the factors associated with MVI in those who underwent liver transplantation.

## Materials and methods

This retrospective study was conducted at the Pakistan Kidney and Liver Institute & Research Center in Lahore from March 2019 to November 2023. Electronic medical records, radiology, and histopathology databases of 523 living donor LT recipients were systematically reviewed. Patients aged 15 years or older with radiological and histopathological diagnoses of HCC on explant liver specimens were included.

Pre-transplant clinical characteristics, including recipient age, gender, co-morbidities, etiology of chronic liver disease, body mass index (BMI), pre-operative alpha-fetoprotein (pre-op AFP) levels, and liver disease severity based on Child-Turcotte Pugh (CTP) class and Model for End-Stage Liver Disease-Sodium (MELD-Na) score were evaluated. Histopathological evaluation of explanted liver specimens included the tumor size, focality, histological grade/differentiation, and MVI. The RETREAT score for each explant specimen was calculated. Pre-transplant tumor burden was assessed using Milan and UCSF criteria based on radiological assessment. Table 1 describes the different parameters utilized by both criteria to select candidates for liver transplantation.

**Table 1:** Milan vs UCSF Criteria for Selecting Hepatocellular Carcinoma Patients for Liver Transplantation

Milan Criteria	University of California San Fransisco (UCSF) Criteria
Solitary tumor ≤ 5cm, or	Solitary tumor ≤ 6.5cm, or
2-3 tumors none exceeding 3cm, and	2-3 tumors none exceeding 4.5cm, with a total tumor diameter of ≤ 8cm, and
No vascular invasion/or extrahepatic spread	No vascular invasion/or extrahepatic spread

Data were analyzed using IBM SPSS Statistics for Windows, Version 20 (Released 2011; IBM Corp., Armonk, New York, United States). Descriptive statistics were computed for continuous variables, and frequencies/percentages were calculated for categorical variables. Univariate analyses, including Mann-Whitney U tests and chi-square tests, were conducted to assess associations between variables and MVI. Logistic regression was employed for multivariate analysis, including variables significant in univariate analysis. Pearson or Spearman correlation coefficients were calculated to examine correlations between continuous variables. Cohen's kappa coefficient was used to measure inter-rater reliability.

## Results

Among the 523 LT recipients, 26% (n=136) were diagnosed with HCC based on pre-transplant radiological imaging and subsequent histopathological evaluation of the explanted liver. Descriptive statistics revealed a mean age of 54.06 ±8.16 (range: 15-70) years, with a male predominance (76.47%, n=104) and hepatitis C virus (HCV) as the primary etiology (72.8%, n=99). The majority of patients had moderately differentiated (grade-II) tumors (75.7%, n=103) and met Milan criteria (74.3%, n=101). Continuous variables displayed no characteristics. The mean body mass index (BMI) was 26.55± 4.36, and the MELD-Na score averaged 14.76 (range: 6- 31). Pre-op AFP levels exhibited a mean of 104.42 ± 308.38 ng/ml. Tumor-related metrics included a mean cumulative tumor size of 4.39 cm, with the largest lesion measuring 8.7 cm.

**Figure 1:**
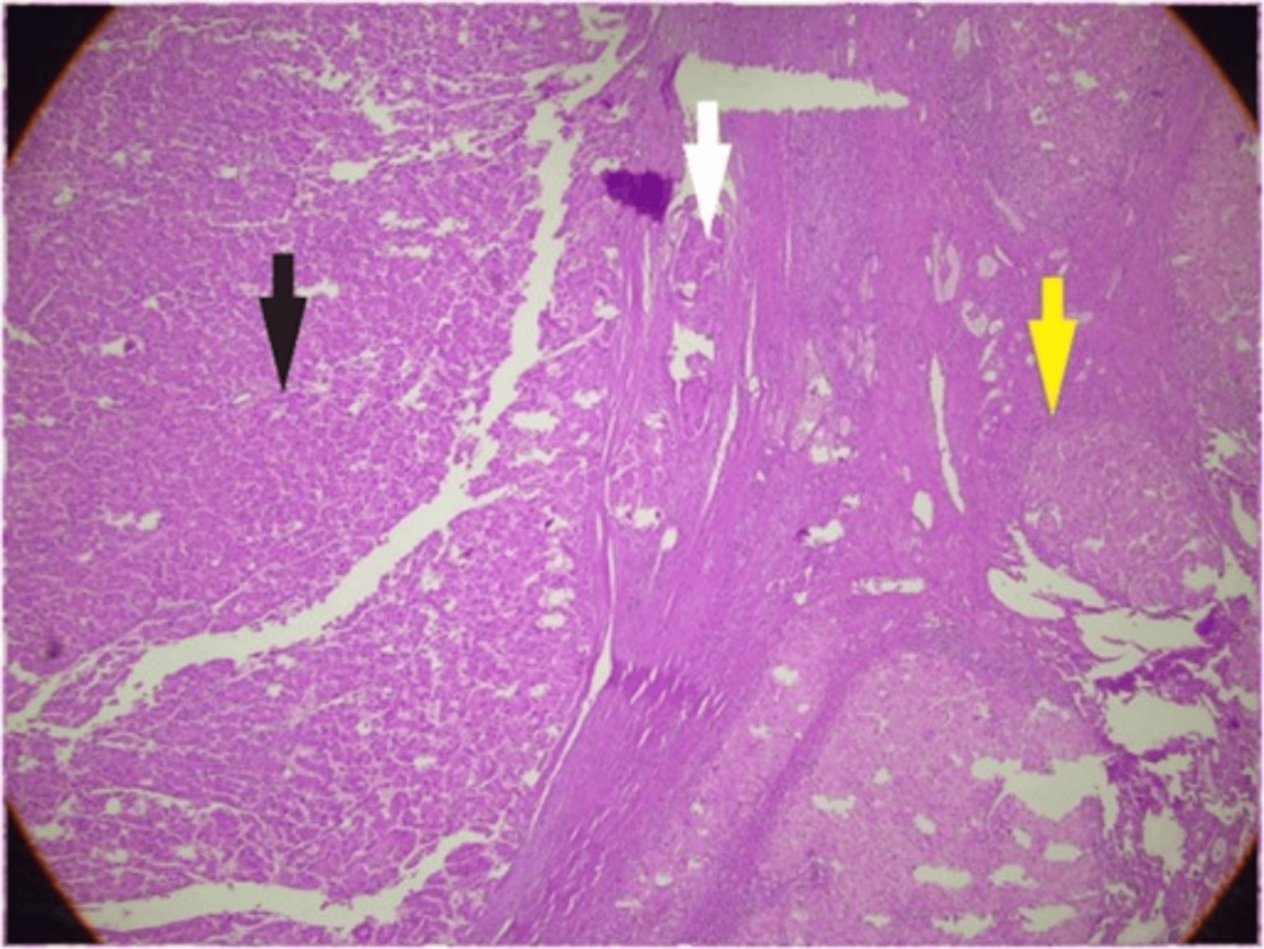
Microvascular Invasion Histopathology image showing microvascular invasion (white arrow) at the interface between hepatocellular carcinoma (black arrow) and nodular, cirrhotic liver (yellow arrow)

Categorical variables highlighted the distribution of key factors. CTP classes A, B, and C constituted 42.6% (n=58), 36.8% (n=50), and 20.6% (n=28), respectively. MVI was reported in 28.7% (n=39) of explant specimens with HCC. Based upon pre-transplant radiological assessment of HCC, solitary tumors were observed in 50.0% (n=68). The median RETREAT score was 2 (range: 1-7) and only 13.97% (n=19) had a score ≥ 5. Figure 1 depicts the histopathological features of MVI.

Univariate analyses utilized non-parametric tests and chi-square tests to explore associations. Notably, pre-op AFP levels (p = 0.001), CTP class (p=0.05), and BMI (p=0.02) were significantly associated with MVI. The frequency of MVI among patients having HCC within and outside Milan criteria was not statistically significant (26.73% vs 34.28%, p = 0.395). Logistic regression was employed for multivariate analysis, incorporating significant variables from univariate analyses. Pre-op AFP was the only independent predictor of MVI (OR: 1.006, 95% CI: 1.003-1.008, p < 0.001). Correlation analysis investigated relationships between continuous variables. A highly significant positive correlation was found between pre-op AFP and cumulative tumor size (p = 0.001). Table 2 highlights the correlation between various factors and MVI.

**Table 2:** Predictors of Microvascular Invasion a: Mann-Whitney U test; b: Chi-square test; *: Represents a statistically significant p-value of <0.05

Univariate Analysis						
Demographic and clinical parameters		Total = 136		Microvascular invasion (MVI) on histopathology		p-value
				Present 39(28.7%)	Absent 97(71.3%)	
		n (%)				
Age (years) (mean ± S. D)				54.49±7.91	53.89±8.304	0.705^a^
Gender; (n, %)	Male	104(76.47%)		27(26%)	77(74%)	0.207 ^b^
	Female	32(23.53%)		12(37.5%)	20(62.5%)	
Etiology of chronic liver disease	Hepatitis B virus	19(14%)		7(36.8%)	12(63.2%)	0.408 ^b^
	Hepatitis C virus	99(72.8%)		29(29.3%)	70(70.7%)	
	Cryptogenic Cirrhosis	8(5.9%)		0(0%)	8(100%)	
	Budd Chiari Syndrome	1(0.7%)		1(100%)	0(0%)	
	Non-alcoholic steatohepatitis	5(3.7%)		2 (40%)	3(60%)	
	Alcoholic cirrhosis	2(1.4%)		0(0%)	2(100%)	
	Hepatitis B virus + Hepatitis D virus	1(0.7%)		0(0%)	1(100%)	
	Progressive familial intrahepatic cholestasis	1(0.7%)		0(0%)	1(100%)	
Body mass index	<18.5	14 (10.3%)		8 (57.1%)	6 (42.9%)	0.02 ^b^,*
	18.5- 24.9	47(34.6%)		13 (27.7%)	34 (72.3%)	
	25-29.9	48(35.3%)		8 (16.7%)	40 (83.3%)	
	≥30	27(19.9%)		10 (37%)	17 (63%)	
Child-Turcotte Pugh (CTP) class	CTP-A	58(42.6%)		23 (39.7%)	35(60.3%)	0.05 ^b^,*
	CTP-B	50(36.8%)		10 (20%)	40 (80%)	
	CTP-C	28(20.6%)		6 (21.4%)	22 (78.6%)	
Tumor grade	Grade-I	21(15.4%)		2 (9.5%)	19 (90.5%)	0.084 ^b^
	Grade-II	103(75.7%)		33 (32%)	70 (68%)	
	Grade-III	3(2.2%)		2 (66.7%)	1 (33.3%)	
	Could not be differentiated	9(6.6%)		2 (22.2%)	7 (77.8%)	
Pre-operative alpha-fetoprotein	≤75 ng/dl	108(79.4%)		25(23.1%)	83(76.9%)	0.005^b^,*
	>75 ng/dl	28(20.6%)		14 (50%)	14(50%)	
Hepatocellular carcinoma focality	Solitary	68(50%)		18 (26.5%)	50(73.5%)	0.912 ^b^
	Two hepatocellular carcinomas (HCCs)	35(25.7%)		12 (34.3%)	23(65.7%)	
	Three HCCs	22(6.2%)		4(18.2%)	18(81.8%)	
	More than three HCCs	11(8.1%)		5(45.5%)	6(54.5%)	
Tumor burden	Yes	101(74.3%)		27(26.7%)	74(73.3%)	0.395 ^b^
Within Milan criteria	No	35(25.7%)		12(34.3%)	23(65.7%)	
Tumor burden beyond Milan and within the University of California San Francisco	Yes	13(9.5%)		5(38.5%)	8(61.5%)	0.412 ^b^
(UCSF)	No	123(90.4%)		34(27.6%)	89(72.4%)	
Tumor burden beyond UCSF	Yes	22(16.2%)		7(17.94%)	15(15.46%)	0.722 ^b^
	No	114(83.8%)		32(82.05%)	82 (84.53%)	
AFP ng/dl (median ±IQR)				29.5(7.06-127.8)	9.31±35.82	0.011 a,*
Model for end-stage liver disease-sodium (MELD-Na) Score (median ±IQR)				14 (12-18)	9.31 (3.9-39.7)	0.705 ^a^
Cumulative tumor size [median ±interquartile range (IQR)]				4.2(2.9-6)	3.8 (2.2-5.7)	0.178^ a^
Size of the largest lesion (median ±IQR)				2.9 (2.4-4.1)	2.6 (1.9-3.5)	0.137^ a^
Multivariate Regression Analysis						
Independent predictors of microvascular invasion			Odds ratio, confidence interval (CI)			p-value
Pre-operative alpha-fetoprotein			OR: 1.006, 95% CI: 1.003-1.008			0.001*

Tumor recurrence was observed in 6.25% (n=8) during a mean follow-up period of 12 months. Only one of them had MVI on the explant specimen. Among recurrent HCCs, 75% (n=6) were within Milan criteria and 50% (n=4) had grade-II tumors on histology. The median pre-op AFP levels were higher among those that had HCC recurrence (22.11ng/dl vs 9.72ng/dl) However, this finding was statistically insignificant (p = 0.275). Radiological and histological findings were concordant in 68.38% (n=93) of cases for HCC staging within the Milan criteria and in 13.23% (n=18) of cases beyond the Milan criteria. Among the remaining discordant results, radiological examination overestimated the tumor burden in 13.97% (n=19) of cases, categorizing them as outside the Milan criteria, whereas histopathological examination confirmed them to be within the Milan criteria. Conversely, it underestimated the tumor burden in 4.41% (n=6) of cases, classifying them as within the Milan criteria, while histopathological examination revealed them to be beyond the Milan criteria. The Cohen’s Kappa (K) for the inter-rater reliability of pretransplant radiological staging and histological staging of HCC was 0.517 (p= <0.001), showing moderate agreement. The sensitivity of pre-transplant radiological assessment to HCC staging according to Milan criteria was 85.5% with a specificity of 73.1% in our cohort. Table 3 presents data on tumor recurrence and the correlation between radiological and histopathological findings for tumor staging. 

**Table 3:** Tumor Recurrence and Radiological vs Histological Correlation * Represents a significant p-value of <0.05

Parameters	Results % (n)
1. Tumor recurrence	6.25% (8)
Microvascular invasion	12.5% (1)
Within Milan	75% (6)
Histological tumor grade II	50% (4)
2. Median Pre-op AFP levels	
Recurrence vs no recurrence	22.11ng/dl vs 9.72ng/dl (p = 0.275)
3. Radiological and histological concordance	
Within Milan	68.38% (93)
Beyond Milan	13.23% (18)
4. Radiological mismatch	
Overestimation	13.97% (19)
Underestimation	4.41% (6)
Inter-rater reliability (IRR)	Kappa =0.517 (p =0.001) *
5. Sensitivity and specificity of radiological assessment for Milan criteria staging	85.5 % and 73.1 %

## Discussion

Surgical treatment options for HCC with the curative intent are resection of an involved liver segment and liver transplantation [11]. However, these options do not ensure recurrence-free survival. Despite extensive pre-transplant workup and application of stringent selection criteria, the risk of HCC recurrence after LT remains a concern. HCC recurrence rates following liver transplantation are around 8-16% [14,15]. Although the Milan criteria is the most widely used criteria to select a patient with HCC for liver transplantation, it only incorporates the morphological details of the tumor (tumor size and number) based on radiological assessments and is unable to predict tumor biology [14]. Discordance exists between the radiological and histological evaluation of HCC, representing another concern for the accurate assessment of tumor morphology on imaging [14]. Pre-transplant tumor burden assessment is underestimated in 20-40% of cases by radiologic modalities [15]. The likely explanation might be the tumor progression while being on the waiting list and the limitation of the radiological imaging to characterize the smaller nodules which make histological evaluation the gold standard for the accurate staging of tumor [15]. In our study, the radiological examination overestimated the tumor burden in 19 (13.97%) cases. Among these overestimated discordant cases, 8(42%) had a history of TACE which may have confounded the evaluation. The tumor burden was underestimated in six (4.41%) patients by the radiological modalities used for staging. The overall sensitivity and specificity for pretransplant radiological modalities to stage HCC according to Milan criteria were 85.5% and 73.1% respectively in our cohort, rendering histopathological examination as the gold standard for accurate staging of HCC.

Various risk factors have been reported in the literature to be associated directly with tumor recurrence after liver transplantation or resection and include high pre-transplant AFP levels, MVI, multiple tumors, large tumor size (>5cm), and a poorly differentiated subtype [16,17]. Several prognostic models that incorporate these parameters have been developed over the last few decades to predict the risk of HCC recurrence after liver transplantation. These include the University of California, Los Angeles (UCLA) nomogram, Pre-Model of Recurrence after Liver Transplantation (MORAL) model, Post MORAL model, Combo MORAL model, RRETREAT, platelet-to-lymphocyte ratio model, R3-AFP score, and AFP model [14]. At our center RETREAT score is used to assess the risk of recurrence for HCC after liver transplantation. Mehta et al. showed that a high RETREAT score increases the risk of HCC recurrence. They showed a recurrence of 1.6% in patients with a RETREAT score of zero and 29% for a score of 5 or higher [13]. In our cohort, only 13.97% (n=19) of explant specimens had a RETREAT score of ≥5 which warrants a vigilant follow-up for early detection of tumor recurrence and optimization of management strategies in this particular group of patients. Except for MVI, all remaining parameters (Serum AFP levels, number of tumors, and largest tumor size) of RETREAT score can be assessed during the pre-transplant phase, therefore predicting MVI before liver transplantation for better patient selection.

MVI is an independent predictor of tumor recurrence and survival after liver transplantation [17]. The superiority of vascular invasion as a better prognostic indicator than conventional selection criteria has been documented in the literature for patients with HCC undergoing either liver resection or transplantation. Lim et al. in a study reported that MVI was a better predictor of tumor recurrence in comparison with Milan criteria among patients undergoing liver resection due to HCC. The study further demonstrated that the post-resection overall survival for patients with HCC beyond Milan criteria but without MVI was comparable to patients with HCC within Milan criteria [18]. Similar outcomes, supporting the superiority of MVI as a better prognostic marker for HCC recurrence after liver transplantation than the conventional selection criteria, were reported in a study from the European liver transplant registry which analyzed a large database of 9324 LT recipients retrospectively [19]. In a retrospective study, Mazzaferro et al. reported that patients transplanted with HCC burden beyond the Up-to-seven criteria had a poor five-year survival rate of 33% when they had MVI, compared to those without MVI, who had a five-year survival rate of 64% [20].

In our study, MVI was present in 28.7% (n=39) of explant liver specimens and the frequency of MVI for recipients with tumor burden beyond Milan criteria was slightly higher (34.28%) as compared to those within the Milan criteria (26.73%) but interestingly this finding was not statistically significant (p= 0.395). After a median follow-up of 12 months, HCC recurrence was observed in 6.25%(n=8) of cases and only 25% of recurrent HCC cases were beyond Milan criteria These results advocate the safety of liver transplantation even when stretching the boundaries beyond conventional Milan criteria.

We aimed to assess the factors that would predict MVI in the pre-transplant period. A comprehensive univariate and multivariate logistic regression analysis revealed AFP as an independent predictor of MVI (OR: 1.006, 95% CI: 1.003-1.008, p < 0.001) in our cohort. Higher pre-transplant AFP levels have been reported to be strongly associated with MVI. McHugh et al. in a study demonstrated that a pre-transplant AFP level above 100 ng/ml was associated with MVI [21]. In our study, the univariate analysis revealed that AFP level > 75ng/ml was significantly associated with MVI (p=0.005). Other factors that have been reported in the literature to be associated with MVI are larger tumor size and higher tumor grade [22]. Tumor focality, grade, individual, and cumulative tumor size did not predict MVI in our cohort.

Tumor biopsy is not performed routinely; however, few studies suggested acquiring a pre-transplant tumor biopsy sample for histopathological analysis of tumor biology for better prognostic stratification [19,22]. The sensitivity of tumor biopsy to identify MVI is only 12.5% and it carries the risk of bleeding and tumor seeding [19]. With advancements in imaging studies and novel radiological techniques, it is now possible to identify radiological features of MVI non-invasively. These include 18 F-FDG PET scans, diffusion-weighted imaging-based habitat imaging, and based radio genomic biomarkers that may be able to accurately predict histological MVI in patients with HCC [19,23,24]. A major limitation of our study is the shorter follow-up period which might otherwise detect higher recurrence rates along with identification of other prognostic markers to predict MVI.

## Conclusions

MVI impacts the overall and recurrence-free survival in patients with HCC undergoing liver transplantation. The pre-transplant identification of MVI is crucial for refining the selection of LT candidates and may serve as a better predictor of recurrence and survival when compared to conventional selection criteria. This study highlights the clinical significance and high relevance of pre-op AFP as a dynamic and simple pre-transplant predictor of MVI in patients with HCC undergoing liver transplantation. This study also advocates for the safety of liver transplantation beyond conventional Milan criteria, promoting extended LT protocols. Newer scoring systems need to be developed to identify patients at risk for MVI. Incorporation of AFP and utilization of novel imaging techniques may offer promising avenues.
